# Spinal and sacroiliac assessment and treatment techniques used by osteopathic physicians in the United States

**DOI:** 10.1186/1750-4732-3-4

**Published:** 2009-04-14

**Authors:** Gary Fryer, Christopher M Morse, Jane C Johnson

**Affiliations:** 1 AT Still Research Institute, AT Still University, Kirksville, MO, USA; 2 Department of Osteopathic Manipulative Medicine, Kirksville College of Osteopathic Medicine, Kirksville, MO, USA; 3 School of Biomedical and Health Sciences, Victoria University, Melbourne, Australia

## Abstract

**Background:**

Osteopathic manipulative medicine texts and educators advocate a range of approaches for physical assessment and treatment, but little is known about their use by osteopathic physicians in the United States.

**Methods:**

A web-based survey using a 5-point Likert scale was developed and e-mailed to 777 practicing osteopathic physician members of the American Academy of Osteopathy. Responses in the "frequently" and "always" categories were combined for reporting purposes. Friedman tests were used to analyze the reported usage of each item. The effect of gender was analyzed using Mann-Whitney tests.

**Results:**

One hundred seventy-one osteopathic physicians completed the survey (22%). For the assessment of spinal somatic dysfunction, paraspinal tissue texture (98%), transverse process asymmetry (89%), and tenderness (85%) were most commonly reported. Myofascial release (78%), soft tissue technique (77%), and patient self-stretches (71%) were most commonly used for treatment of the spine. For assessment of pelvic landmark asymmetry, the anterior superior iliac spine (ASIS, 87%), sacral base (82%), posterior superior iliac spine (81%), sacral sulci (78%), iliac crests (77%), and inferior lateral angle of the sacrum (74%) were commonly palpated. For assessment of sacroiliac joint motion, ASIS compression (68%) was most commonly used. Sacroiliac pain provocation tests were also employed although their use was less common than asymmetry or motion tests. Muscle energy (70%), myofascial release (67%), patient self-stretches (66%), osteopathy in the cranial field (59%), muscle strengthening exercises (58%), soft tissue technique (58%), and articulatory technique (53%) were most commonly used for treatment of the pelvis and sacroiliac. The effect of gender was significant for many of the treatment procedures, with females using more soft tissue and muscle energy and males more high-velocity techniques. The majority of respondents document the types of osteopathic manipulative techniques used (83%), document somatic dysfunction with Fryette nomenclature (64%), and bill for osteopathic manipulative treatment (92%).

**Conclusion:**

Respondents reported the use of a broad range of assessment and treatment approaches. Results suggest a higher use of myofascial release and cranial technique and lower use of high-velocity techniques in this group of physicians compared to previous studies.

## Background

Osteopathic practice typically involves a holistic, multidimensional approach to patient care and may include consideration of emotional, psychobehavioral, environmental, ergonomic, and biomechanical issues. Physical examination of patients may commonly be performed in relation to the osteopathic concept of somatic dysfunction. Somatic dysfunction has been described as a functional disturbance to tissues of the musculoskeletal system and related vascular and neurological components, amenable to osteopathic manipulation [1].

Authors in the field of osteopathic manipulative medicine (OMM) have proposed a wide range of methods and approaches for the assessment and treatment of spinal and pelvic somatic dysfunction. There are at least 28 osteopathic techniques listed in the *Glossary of Osteopathic Terminology *[2] which, despite their overlap, can be grouped into direct, indirect, and combined techniques. These are all typically used within the context of the various conceptual models for osteopathic manipulative treatment (OMT), including the postural/biomechanical, neurological and respiratory/circulatory models [1,3-7]. In contrast to authors outside the United States (US) [8-10], many American authors of osteopathic texts [1,5,6,11] base structural assessment and treatment of the spine on the biomechanical model proposed by Fryette [12] and base pelvic approaches on the biomechanical model proposed by Mitchell [13]. Little is known, however, about the OMM preferences of osteopathic physicians regarding assessment and treatment approaches or documentation and billing practices. Few studies have been conducted to determine which approaches are commonly used in OMM practice, either in the US or elsewhere, and it is therefore difficult to compare the practice of OMM throughout the world.

Johnson and Kurtz [14] surveyed primary care osteopathic physicians who were members of the American Osteopathic Association for preferences regarding OMT selection from a list of techniques in *Foundations for Osteopathic Medicine *[7]. Respondents were more likely to use direct techniques, such as soft tissue, high-velocity low-amplitude thrust (HVLA), and muscle energy, than indirect techniques. Female physicians and older respondents, however, were more likely to use indirect approaches than younger male respondents. OMT specialists were more likely to use a broader range of techniques, including cranial and fascial ligamentous release techniques. This study did not survey physicians about their preferences regarding diagnostic methods.

Few studies have examined OMT preferences in other countries. In 2004, the Australian Osteopathic Association conducted a member-wide census, which included a survey with a snapshot component [15,16]. Osteopaths estimated that they more frequently used direct techniques [16], and this opinion was supported by the snapshot survey of actual usage that indicated the most frequently used techniques were soft tissue (71% of patients), articulation (57%), HVLA (51%), and muscle energy (50%) [15]. The census did not examine diagnostic methods. Peace and Fryer surveyed the Australian profession for pelvic and sacroiliac diagnostic approaches and found that most respondents used procedures consistent with the Mitchell model, such as flexion tests and identification of pelvic landmark asymmetry, but supported these tests with additional motion and pain provocation tests [17].

In the United Kingdom (UK), the General Osteopathic Council conducted a snapshot survey to update information about the practice of osteopathy obtained from previous snapshots [18]. The survey requested information about patients and practitioners, including the treatment techniques employed on the snapshot day. Results were similar to the surveys conducted in the US and Australia: osteopaths most frequently used direct techniques, particularly soft tissue stretching (78% of patients), articulation (75%), HVLA (47%), and muscle energy (26%).

Despite the variety of OMM approaches advocated, only one study [14] has surveyed the American osteopathic profession regarding the use of different OMM techniques and none has investigated the diagnostic methods currently in use. Through the use of a web-based survey, the present study investigated methods used for the assessment and treatment of spinal and pelvic somatic dysfunction by osteopathic physicians. Additionally, the documentation and billing of OMT services by osteopathic physicians were investigated. Given that the usage of OMT within the American profession is reportedly low [19,20], practicing physician members of the American Academy of Osteopathy (AAO), an association which emphasizes the integration of osteopathic principles and manipulative treatment in patient care, were targeted for this study.

## Methods

The subjects of this study were practicing AAO physician members. Members who were students, interns, residents, or international affiliates were excluded from the survey distribution list to ensure that only practicing AAO physicians responded.

A web-based survey with a 5-point Likert scale was developed for the study. The survey contained questions pertaining to respondent demographics, procedures used to identify spinal somatic dysfunction, knowledge and use of spinal biomedical models, use of diagnostic imaging before OMT, techniques used to treat spinal somatic dysfunction, procedures used to identify pelvic and sacroiliac somatic dysfunction, techniques used to treat pelvic and sacroiliac somatic dysfunction, and documentation of and billing practices for somatic dysfunction and OMT (see Additional file 1). Open-ended "Other" responses were included in the survey for those procedures not listed as a choice.

Prior to dissemination of this survey, a draft survey was piloted on five practicing osteopathic physicians and three OMM residents. Based on their suggestions, the survey was slightly modified. Of the 1625 eligible AAO members, 777 (48%) had listed e-mail addresses, and an invitation to participate with a web link to the survey was e-mailed to these members. Two weeks after this initial mailing, a reminder e-mail was sent. The local institutional review board approved the study.

The available response categories (strongly disagree, disagree, etc.) are ordinal and were converted into numerically weighted scales. For reporting purposes, the combined percentage of responses in the "Frequently" and "Always" categories were combined. Open-ended responses in the "Other" category were examined and redundant or inappropriate responses (such as methods for global structural assessment rather than the requested segmental approaches) were excluded. Friedman tests were used to determine if significant differences existed between the reported usage of each item. The effect of gender and years of practice (categorized by decade) on preference of assessment and treatment techniques were analyzed using Mann-Whitney tests.

## Results

### Participants

One hundred seventy-two osteopathic physicians responded to the survey, a 22% response rate. One respondent was excluded for reporting no use of OMT. Sixty-nine percent of the respondents were male, had a mean of 15 years experience (SD = 11, range 0–53), and were educated at a variety of schools. The most common training institutions were Kirksville College of Osteopathic Medicine (KCOM), New York College of Osteopathic Medicine (NYCOM) and University of New England College of Osteopathic Medicine (UNECOM); each comprised 11% of respondents, with the remaining 67% of respondents having trained at 17 other osteopathic colleges.

Respondents indicated that they used OMT: 59% estimated they used OMT on 76–100% of patients, 13% on 51–75%, 14% on 26–50%, and 15% on 1–25%. Listed specialties or designations were OMM/Neuromusculoskeletal Medicine (60%), Family Practice/OMT (51%), Fellow of AAO (12%), Sports Medicine (6%), Physical Medicine and Rehabilitation (5%), Medical Acupuncture (2%), Emergency Medicine (2%), and Other (13%).

### Assessment of spinal dysfunction

**Figure 1:**
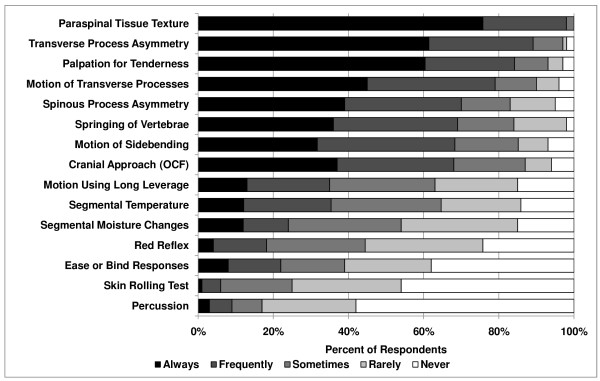
**Procedures used to identify spinal somatic dysfunction**.

Osteopathic physicians reported the most common use of procedures associated with the structural approach (between 69% and 98%), including palpation of paraspinal tissue texture (98%), transverse process asymmetry (89%), and tenderness (85%) [*P *< .0001, (Figure 1)]. The assessment of motion of transverse processes (80%), spinal processes asymmetry (70%), springing of vertebrae (69%), motion of sidebending (69%), and use of osteopathy in the cranial field (OCF, 68%) was also commonly reported. The skin rolling test (6%) and percussion (9%) were least commonly used. Nineteen responses were included in the "Other" category, the most frequent being Jones points/counterstrain, lateral translatory or oscillatory maneuvers, and energetic based procedures.

There were no significant differences between males and females on the procedures used. Osteopathic physicians in practice for 10–19 years were more likely to use springing of vertebrae than 0–9 years and 20+ years (*P *= .02, 79% vs. 67% and 65%, respectively).

Most respondents had knowledge of the Fryette spinal biomechanical model (89%). Sixty percent reported that the model was frequently/always useful for diagnosis and treatment although 21% indicated they commonly diagnosed motion restriction combinations that contradicted the model. The use of radiology and diagnostic imaging was low, with only 7% of respondents frequently/always using imaging prior to delivering cervical OMT and with 3% using imaging prior to delivering OMT to other regions of the spine.

### Treatment of spinal dysfunction

**Figure 2:**
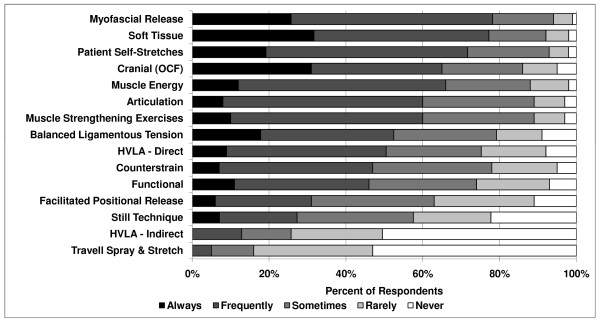
**Treatment of spinal dysfunction**.

Procedures for the treatment of somatic dysfunction of the spine reported to be most commonly used were myofascial release (direct or indirect, 78%), soft tissue technique (77%), patient self-stretches (71%), and OCF (65%) (Figure 2). Forty-six responses for spinal treatment techniques were volunteered under "Other" and included the use of the percussion hammer, visceral technique, acupuncture, and prolotherapy.

There was a significant effect of gender for many of the procedures. Female respondents reported more common use of soft tissue technique (*P *= .003, 91% of females vs. 71% of males), muscle energy technique (*P *= .03, 79% vs. 60%), and patient strengthening exercises (*P *= .04, 81% vs. 67%). Male respondents reported more common use of direct HVLA (*P *= .01 58% of males vs. 34% of females). Osteopathic physicians in practice for 0–9 years were more likely to use the Still technique than 10–19 years and 20+ years (*P *= .02, 34% vs. 20% and 28%, respectively).

### Assessment of pelvic and sacroiliac dysfunction

**Figure 3:**
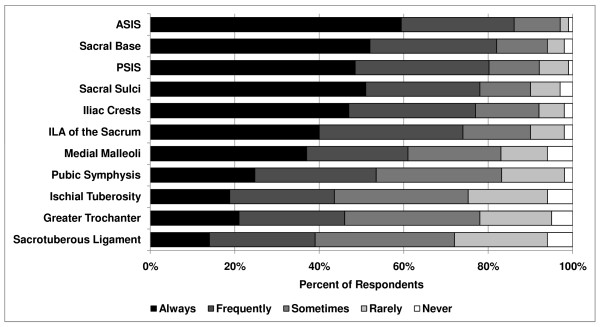
**Pelvic landmarks assessed for asymmetry**.

Between 39% and 87% of respondents estimated that they frequently/always assessed various pelvic landmarks for bony asymmetry. The most common landmarks assessed were the anterior superior iliac spine (ASIS [87%]), sacral base (82%), posterior superior iliac spine (PSIS [81%]), sacral sulci (78%), iliac crests (77%), and inferior lateral angle of the sacrum (74%) [*P *< .0001, (Figure 3)]. Twenty responses listed under "Other" included assessment of L5, pelvic and lumbar musculature, and iliolumbar ligaments.

**Figure 4:**
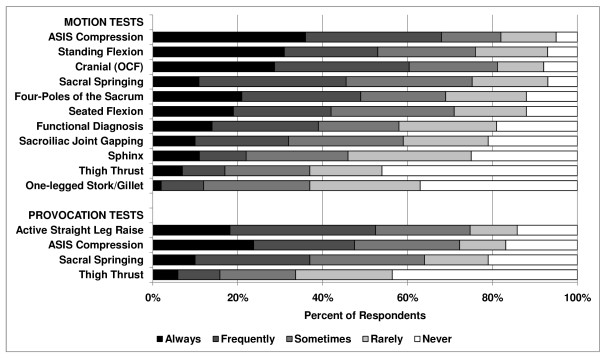
**Sacroiliac motion & pain provocation tests**.

Sacroiliac joint (SIJ) motion tests were also commonly used. The most common motion test was ASIS compression (68%), followed by OCF (61%), the standing flexion test (54%), and sacral springing (46%) [*P *< .0001, (Figure 4)]. The one-legged stork/Gillet test (12%), thigh thrust (17%), and Sphinx test (23%) were least commonly used. Fifteen responses for motion tests not listed in the survey but volunteered under "Other" included Patrick's FABER, Gaenslen, and Trendelenburg tests, as well as many other procedures only reported in single instances.

The most commonly used SIJ pain provocation tests were the active straight leg raise (52%) and ASIS compression (48%), but these tests were less employed than many of the landmark and motion tests [*P *< .0001, (Figure 4)]. In fact, five respondents volunteered that they deliberately do not use pain provocation tests. Eighteen responses for provocation tests not listed in the survey but volunteered under "Other" included Patrick's FABER test and deep pressure on the sacral sulci.

Female respondents more commonly chose to palpate the ASIS (*P *= .046, 89% of females vs. 86% of males), sacral sulci (*P *= .02, 87% vs. 75%), sacral base (*P *= .01, 92% vs. 78%), iliac crest height (*P *= .04, 87% vs. 74%), and medial malleoli (*P *= .03, 70% vs. 57%); and to assess dysfunction using the standing flexion test (*P *= .007, 66% vs. 48%), seated flexion test (*P *= .02, 54% vs. 37%), ASIS compression test (*P *= .04, 74% vs. 65%), or the ASIS compression test for pain provocation (*P *= .04, 54% vs. 45%). There was no significant effect of years in practice on assessment of pelvic and sacroiliac dysfunction.

### Treatment of pelvic and sacroiliac dysfunction

**Figure 5:**
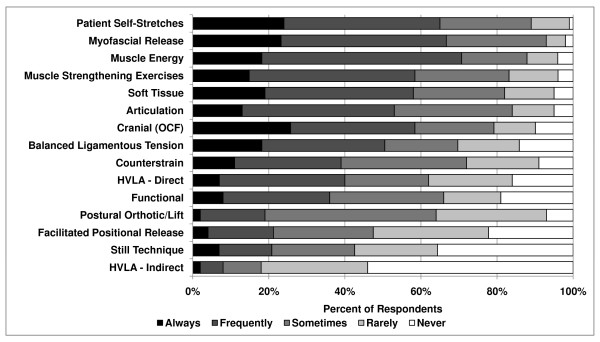
**Treatment of pelvic and sacroiliac dysfunction**.

Procedures for the treatment of pelvic and SIJ somatic dysfunction frequently/always used were muscle energy (70%), myofascial release (67%), patient self-stretches (66%), OCF (59%), muscle strengthening exercises (58%), soft tissue technique (58%), and articulatory technique (53%) [*P *< .0001, (Figure 5)]. Indirect HVLA was least commonly used (8%). Twenty-two responses for treatment procedures not listed in the survey but volunteered under "Other" included the use of the percussion hammer, neuromuscular reeducation exercises, prolotherapy, and acupuncture.

As for spinal treatment, there was also a significant effect of gender for treatment of the pelvis and sacroiliac joint. Female respondents reported more common use of soft tissue technique (*P *= .003, 69% of females vs. 53% of males), articulation (*P *= .02, 63% vs. 49%), myofascial release (*P *= .03, 76% vs. 62%), Still technique (*P *= .04, 27% vs. 18%), prescriptions for patient self-stretching (*P *= .005, 75% vs. 61%), and muscle strengthening exercises (*P *= .03, 66% vs. 54%). Male respondents reported more common use of HVLA techniques (*P *= .006, 47% of males vs. 21% of females). Osteopathic physicians in practice for 0–9 years were more likely to use balanced ligamentous tension than 10–19 years and 20+ years (*P *= .03, 63% vs. 43% and 42%, respectively).

### Documentation and billing of OMM

**Figure 6:**
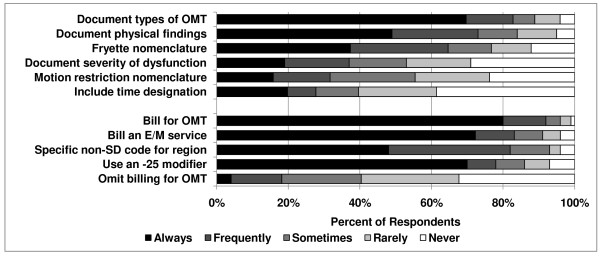
**Documentation and billing for OMT**.

Most respondents documented spinal somatic dysfunction using the Fryette nomenclature, where apparent position or motion preference are recorded using an abbreviation (e.g., T5 ERS_R_, E for extended, R for rotated, S for sidebent) (64%), rather than using motion restriction nomenclature (e.g., T5 restricted rotation right, side bending left) (32%). The majority of respondents (82%) documented the types of OMT used (e.g., HVLA, muscle energy, etc.). Most respondents also documented the physical findings (e.g., right transverse process posterior, positive left seated flexion test) associated with spinal somatic dysfunction (73%), but few documented the time required to perform OMT (27%) (Figure 6).

The vast majority of respondents reported that they always/frequently billed for OMT (92%) (Figure 6). Additionally, they also billed for an evaluation and management (E&M) service (84%), used a specific non-somatic dysfunction code for the region of complaint (82%), and used a -25 modifier (78%, allowing bundling of billing for significant, separately identifiable evaluation and management service by the same physician on the same day of the procedure).

## Discussion

As in a previous study [14] aimed at the profession in the US, osteopathic physicians in the present study used a wide range of techniques for treatment of spinal and pelvic somatic dysfunction. Respondents indicated a preference for soft tissue based techniques and – compared to studies from other countries – a greater preference for cranial methods. This utilization of OCF was higher than expected, based on the previous American survey [14] and studies of osteopaths in the UK and Australia [15,18]. Survey responses also suggest a gender bias for some of the treatment techniques.

The reported preference of survey respondents for cranial techniques is interesting. Johnson and Kurtz [14] reported that OCF was ranked last on a list of manual techniques used by osteopathic physicians. In that survey, however, those respondents who listed OMT as a specialty used a broader range of techniques and had a significantly greater preference for OCF than those who did not list OMT as a specialty [14]. Perhaps the similarity of this subgroup with our survey respondents explains the similar preference for cranial approaches. In contrast, osteopaths in the UK and Australia (where OMT is the primary treatment modality) [15,18] seem less inclined to use OCF. Snapshot studies in those countries report cranial approaches are used on 23% of patients, but those studies also report what techniques were used for patients over a full treatment. Therefore, a treatment specific to spinal or pelvic dysfunction would likely involve even less use of cranial techniques because OCF is emphasized for dysfunction in the "involuntary mechanism" (the mechanism postulated to underlie the cranial rhythmic impulse) and may be used to assess different phenomena than biomechanical function of the spine and pelvis.

The Fryette model of spinal coupled motion was commonly used by respondents. This result is expected since American osteopathic textbooks advocate assessment based on these principles [1,5,6,11]. The use of the Fryette model by osteopaths in other countries has not been examined, but it is expected to be less given that many authors from other countries have not used this model [8-10]. The Fryette model has been criticized for its prescriptive diagnostic labeling and questionable inferences concerning motion restriction from static positional assessment [21,22]. Recent studies suggest that spinal coupled motions are inconsistent and there is variability between spinal levels and between individuals for motion in the lumbar, thoracic, and, to a lesser extent, cervical spine [22-28]. Lack of consistency in coupled spinal motion should be a concern to those who advocate the Fryette model as a means of predicting triplanar motion restrictions. Given the common use of this model and its endorsement from the Educational Council of Osteopathic Principles (ECOP) [29], which guides the curriculums of OMM programs, the American profession may wish to re-examine the validity and usefulness of the Fryette model.

Reported preferences for assessment of the pelvis and sacroiliac joints were consistent with the biomechanical and treatment model proposed by Mitchell [13] and advocated by most American osteopathic texts [1,5,6,11]. The Mitchell model recommends the use of motion tests (typically the flexion tests) to determine the side of the dysfunction and the identification of landmark asymmetry to determine the type of dysfunction. The positive responses for assessment of symmetry of pelvic landmarks in the present study were similar to an Australian study, suggesting that this model is also prevalent outside the US [17]. Motion testing of the sacroiliac joint in the present study was commonly performed, and cranial diagnosis for the pelvis and sacroiliac joint was also popular. The Australian study reported frequent use of motion tests, but a considerably lower use of cranial diagnosis (30% compared to 61% in the present study) [17].

Sacroiliac joint pain provocation tests, procedures intended to reproduce the patient's familiar pain by stressing the sacroiliac joint and thus implicating it as a pain generator, were used by just over half the respondents. The most popular test, the active straight leg raise, is rarely referred to in the osteopathic literature, and some respondents may have confused it with the straight leg raise for nerve root irritation. ASIS compression (performed in a similar way to the motion test but intended to provoke the complaint) was also reportedly used by nearly half of respondents. These two tests are rarely mentioned in osteopathic texts, but their reliability and validity are supported by the scientific literature [30,31]. Given these circumstances, it is interesting that they are used by a substantial proportion of respondents. An Australian survey of osteopaths found a comparable use of pain provocation tests [17], which suggests that both groups use a pragmatic approach to patient care that includes procedures other than those typically recommended by the profession.

Female respondents reported more frequent use of soft tissue technique, muscle energy, and strengthening exercises for treatment of the spine and pelvis, whereas male respondents more frequently used HVLA. Similarly, Johnson et al [14] found that female respondents were more likely than men to use indirect techniques. These gender preferences may reflect the physical strength required to perform direct techniques, such as HVLA. Alternatively, it is possible that the patient populations seen by male and female practitioners are different, and that the patients seen by female practitioners (who may comprise more women and children) may prefer and request more gentle treatment approaches.

While most respondents in the present study reported that they document physical findings and the type of OMT delivered, a substantial minority do not frequently document their findings or treatment. This represents a potential concern for the profession given the importance of maintaining accurate records of treatment, particularly in the event of adverse reactions or litigation. The common use of the Fryette spinal model explains the documentation of somatic dysfunction using this model's positional notation. Most respondents bill for OMT and use a -25 modifier. By using the -25 modifier, both the patient visit and OMT can be billed at the same visit, providing an economic incentive for OMT use. These findings suggest a healthy amount of business savvy amongst this group and an awareness of efficient billing practices for OMT. Respondents reported a low use of diagnostic imaging prior to OMT, which is consistent with current guidelines that state plain imaging is of little use for non-specific spinal pain [32].

This study has a number of limitations. The response rate to the survey was relatively low and generalizing these results to the entire AAO membership may be inappropriate. However, according to data provided by the AAO, the demographics of the sampled respondents closely reflect the membership as a whole. Respondents and membership were strikingly similar for gender (69% and 60% male, respectively) as was place of osteopathic training, with KCOM, UNECOM, and NYCOM listed as the most common institutions for both the study respondents and AAO membership (each institution accounting for 11% of respondents compared to 13%, 10%, and 9% of the total membership). Study respondents listed OMM/Neuromusculoskeletal Medicine (60%) and Family Practice/OMT (51%) as their most common specialties, which were also the most common specialties for the AAO membership (36% and 43%, respectively, followed by a list of other specialties which each accounted for less than 5% of members). Although a higher percentage of study respondents than AAO members reported their specialties as OMM/Neuromusculoskeletal Medicine and Family Practice/OMT, this increase may be a result of limiting the survey to only those members who use OMT and of the exclusion of students, interns, and residents. No information was available on years of practice experience for members of the AAO, but the comparisons above support the study sample as being representative of the entire membership.

Members of the AAO were targeted because they have an expressed interest in OMM. Because the use of OMT is diminishing within the osteopathic profession, study respondents do not necessarily represent the broader profession. Additionally, due to the relatively low response rate for the survey, a sample bias may favor more computer savvy respondents. Those practitioners who did not respond to the email invitation or did not have an email address listed with the AAO may potentially have been less proficient in newer methods and, thus, have different treatment preferences. Therefore, caution is necessary when generalizing the results to the membership of the AAO even though similar demographics support this group as representative. Researchers have recommended email and web-based surveys as holding great promise as a fast, inexpensive medium for health research, but comparisons between survey delivery methods have so far demonstrated a greater response rate to postal surveys [33,34]. A combined postal and email survey would likely have improved the response rate, and this method should be considered for future surveys until the time the growing internet culture favors a better response rate for electronic distribution.

It should be understood that this study surveyed what respondents *said *they did in practice and was not a snapshot survey or a record of what approaches respondents actually used in practice. The responses are therefore subject to recall bias of the practitioners. Although requiring more resources, a snapshot survey is a worthwhile addition for future studies to provide a more accurate indication of technique preferences in the practice setting.

Although the intention of this study was to determine what treatment approaches osteopathic physicians use, a review of the validity and reliability of these approaches may provide additional context for interpreting study results. For instance, few procedures for segmental dysfunction – with the exception of palpation for tenderness and pain provocation – have acceptable interobserver reliability [33]. For assessment of pelvic and sacroiliac dysfunction, landmark asymmetry and tests that assess sacroiliac motion have been criticized for poor reliability and lack of validity [31,34-36]. While cranial approaches for the diagnosis and treatment of the spine and pelvis were favored by study respondents, their use is debated within the profession, with criticisms ranging from the biological plausibility of the concept to the lack of examiner reliability and outcome studies [37,38]. Using the results from this study, which provides information about tests that are in common use, future studies could be designed to improve the reliability of these procedures. Alternatively, the osteopathic profession may want to reconsider the use of these tests.

## Conclusion

Osteopathic physician members of the AAO who responded to our web-based survey reported using a broad range of osteopathic diagnostic and treatment methods. Their responses were consistent with the Fryette spinal model and Mitchell pelvic model, both commonly advocated by American osteopathic textbooks and educational institutions. Respondents preferred soft tissue treatment approaches for both spinal and pelvic dysfunction, as well as cranial approaches. The strong inclination for cranial techniques was higher than reported in previous American and international studies and may reflect the preferences of osteopathic physicians who specialize in OMT.

## Competing interests

GF is an associate member of the American Academy of Osteopathy.

## Authors' contributions

GF devised the research concept and design, developed the survey instrument, implemented the survey, assisted in analysis of data, and wrote the manuscript. CM assisted with survey development and writing of the manuscript. JJ assisted with research concept and design, analyzed the data, and assisted with writing the manuscript.

## Supplementary Material

Additional file 1 **Survey instrument**. web survey. Click here for file
