# Supplementary material for: Spinal and sacroiliac assessment and treatment techniques used by osteopathic physicians in the United States
Source: Osteopath Med Prim Care. 2009 Apr 14;3:4. doi: 10.1186/1750-4732-3-4 (PMC2676310; doi:10.1186/1750-4732-3-4)
Supplement: Additional file 1 — Survey instrument. web survey. [file 1750-4732-3-4-S1.doc]

# Additional file 1: Survey instrument

Note: this appendix does not contain the formatting of the original web survey

**Spinal and Sacroiliac Joint Assessment within the American Osteopathic Profession**

1. Osteopathic / Medical School attended:
2. Graduation Year:
3. Years in practice:
4. Gender:
5. Percentage of patients on which you perform Osteopathic Manipulative Treatment (OMT):

- 0%
- 1-25%
- 26-50%
- 51-75%
- 76-100%)

1. Specialty(ies) or Designation … Indicate all that apply:

- NMM/OMM or C-SPOMM
- FAAO
- FP/OMT
- PM&R
- Orthopedics
- Sports Medicine

Other (please specify):

**SECTION 1: SPINAL DIAGNOSIS**

The following questions relate to the assessment of SPINAL somatic dysfunction.

1. Please indicate how ***commonly*** you use the following procedures to ***identify spinal somatic dysfunction***:

Scale: a) Never b) Rarely c) Sometimes d) Frequently e) Always

- Palpation of Paraspinal Tissue Texture
- Skin Rolling Test
- Palpation for Tenderness
- Observation for Red Response (Red Reflex; erythema following manual stroking)
- Palpation for Segmental Temperature Variation
- Palpation for Segmental Moisture Changes (sweating)
- Palpation of Transverse Process Asymmetry
- Palpation of Spinous Process Asymmetry
- Springing of the Vertebrae for Restriction (posterior – anterior pressure)
- Palpation of the Motion of Transverse Processes (vertebral rotation or sidebending using direct contact on the transverse process)
- Palpation of the Motion of Sidebending Using Direct Contact over Articular Pillars (medial translatory motion)
- Palpation of the Motion Using Long Leverage (e.g. inducing trunk rotation by moving patient’s elbows, while monitoring the vertebral motion; inducing lumbar flexion by bending the hips and knees, while monitoring the vertebral motion)
- Percussion (from Johnson functional method)
- Palpation of Ease or Bind Responses at Motion Induction (Hoover or Johnson functional methods)
- Palpation of Cranial Rhythmic Impulse or Primary Respiratory Mechanism

1. If you commonly use any other procedures to identify spinal somatic dysfunction please specify.
2. The following statements relate to knowledge and use of models of spinal biomechanical models

Scale: a) Strongly disagree b) Disagree c) Neutral d) Agree e) Strongly agree

- I am knowledgeable of the Fryette model of spinal coupling, type 1 and 2 coupled spinal motions
- I diagnose and/or name spinal somatic dysfunctions according to the Fryette model (e.g. neutral & non-neutral dysfunctions, type 1 neutral (NSR) and type 2 non-neutral (FSR & ERS) coupled motion)
- The Fryette model is useful and reliable for my diagnosis and treatment
- I identify motion restrictions that contradict the Fryette model (e.g. restriction of flexion *and* type 1 rotation/sidebending coupling; restriction of type 2 rotation/sidebending coupling *without* flexion or extension involvement)
- I identify motion restrictions that contradict commonly taught motion models (e.g. typical cervical segments sidebending and rotating in opposite directions)

1. Diagnostic imaging

Scale: a) Never b) Rarely c) Sometimes d) Frequently e) Always

- How commonly do you use radiology/imaging prior to delivering cervical OMT?
- How commonly do you use radiology/imaging prior to delivering OMT elsewhere in the spine?

**SECTION 2: SPINAL TREATMENT**

The following questions relate to the treatment of SPINAL somatic dysfunction.

1. Please indicate how ***commonly*** you use the following procedures to ***treat spinal somatic dysfunction***:

Scale: a) Never b) Rarely c) Sometimes d) Frequently e) Always

- Soft Tissue Techniques
- Articulation Technique (passive joint mobilization)
- Myofascial Release Techniques (Direct, Indirect, or Combined)
- High Velocity, Low Amplitude thrust (HVLA) -- Direct
- High Velocity, Low Amplitude thrust (HVLA) – Indirect (Maigne) Method
- Muscle Energy Technique
- Functional (indirect) technique
- Counterstrain
- Facilitated Positional Release
- Balanced Ligamentous Tension or Ligamentous Articular Strain
- Still Technique
- Cranial Technique (Osteopathy in the Cranial Field)
- Travell Spray & Stretch
- Prescription for Patient Self-Stretches
- Prescription for Patient Muscle Strengthening Exercises

1. If you commonly use any other procedures to treat spinal somatic dysfunction please specify.

**SECTION 3: PELVIC & SACROILIAC DIAGNOSIS**

The following questions relate to the assessment of PELVIC & SACROILIAC somatic dysfunction.

*A) PALPATION OF LANDMARKS FOR ASYMMETRY*

1. Please indicate how ***commonly*** you ***palpate for asymmetry*** of the following landmarks in order to identify pelvic and sacroiliac somatic dysfunction:

Scale: a) Never b) Rarely c) Sometimes d) Frequently e) Always

- Anterior Superior Iliac Spines (ASIS)
- Pubic Symphysis
- Posterior Superior Iliac Spines (PSIS)
- Sacral Sulci
- Sacral Base
- Inferior Lateral Angles (ILA) of the Sacrum
- Iliac Crests
- Ischial Tuberosity
- Sacrotuberous Ligament
- Greater Trochanter
- Medial Malleoli

1. If you commonly palpate other landmarks for asymmetry in order to identify pelvic and sacroiliac somatic dysfunction please specify.

*B) MOTION TESTS*

1. Please indicate how ***commonly*** you use the following ***sacroiliac motion tests*** in order to ***identify pelvic and sacroiliac somatic dysfunction***:

Scale: a) Never b) Rarely c) Sometimes d) Frequently e) Always

- Standing Flexion Test
- Seated Flexion Test
- One-legged Stork Test / Gillet Test
- Anterior Superior Iliac Spine (ASIS) Compression Test
- Motion Testing of the Four-Poles of the Sacrum
- “Thigh thrust” (Supine, hip flexed 90^0^, compression through femur)
- Sacral Springing – patient prone
- Sacroiliac Joint Gapping using internal hip rotation and/or adduction as lever (patient prone)
- Sphinx Test (‘Backward Bending Test’; palpation of sacral base in the sphinx position)
- Functional Diagnosis
- Cranial Diagnosis (Osteopathy in the Cranial Field)

1. If you commonly use any other sacroiliac motion tests in order to identify pelvic and sacroiliac somatic dysfunction please specify.

*C) PAIN PROVOCATION TESTS*

1. Please indicate how ***commonly*** you use the following ***sacroiliac pain provocation tests*** (tests intended to reproduce the patent’s familiar pain) in order to ***identify pelvic and sacroiliac somatic dysfunction***:

Scale: a) Never b) Rarely c) Sometimes d) Frequently e) Always

- Anterior Superior Iliac Spine (ASIS) Compression Test
- “Thigh Thrust” (Supine sacroiliac springing, hip flexed, compression through femur)
- Sacral Springing – patient prone
- Active Straight Leg Raise

1. If you commonly use any other sacroiliac pain provocation tests in order to identify pelvic and sacroiliac somatic dysfunction please specify.

**SECTION 4: PELVIC & SACROILIAC TREATMENT**

The following questions relate to the treatment of PELVIC & SACROILIAC somatic dysfunction.

1. Please indicate how ***commonly*** you use the following procedures to ***treat pelvic and sacroiliac somatic dysfunction***:

Scale: a) Never b) Rarely c) Sometimes d) Frequently e) Always

- Soft Tissue Techniques
- Articulation (passive joint mobilization)
- Myofascial Release Techniques (Direct, Indirect, or Combined)
- High Velocity, Low Amplitude thrust (HVLA) -- Direct
- High Velocity, Low Amplitude thrust (HVLA) – Indirect (Maigne) Method
- Muscle Energy Technique
- Functional (indirect) technique
- Counterstrain
- Facilitated Positional Release
- Balanced Ligamentous Tension or Ligamentous Articular Strain
- Still Technique
- Cranial Technique (Osteopathy in the Cranial Field)
- Prescription for Patient Self-Stretches
- Prescription for Patient Muscle Strengthening Exercises
- Prescription or Fitting of Postural Orthotic / Lift

1. If you commonly use other procedures to treat pelvic and sacroiliac somatic dysfunction please specify.

**SECTION 5: DOCUMENTATION & BILLING**

The following questions relate to the *documentation (recording clinical notes) and billing* for the diagnosis of somatic dysfunction and treatment with osteopathic manipulative treatment (OMT)

1. Please indicate how ***commonly*** you do the following:

Scale: a) Never b) Rarely c) Sometimes d) Frequently e) Always

- Document the *physical findings* associated with spinal somatic dysfunction (e.g. Right transverse process posterior, positive left seated flexion test)
- Document the severity of the somatic dysfunction using a numerical (e.g. 0-3) or narrative (mild-severe) rating scale
- Document the specific diagnosis of spinal somatic dysfunction using Fryette nomenclature (e.g. T5 ERS_R_) of apparent position or motion preference
- Document the specific diagnosis of spinal somatic dysfunction using motion restriction nomenclature (e.g. T5 restricted rotation right, sidebending left)
- Include a specific non-somatic dysfunction code for the region treated with OMT (e.g. lumbar strain, myalgia)
- Bill for performing OMT
- Omit billing for OMT in one or more treated regions that had only minor or compensatory somatic dysfunction
- Bill an E&M (examination & medical) service when you evaluate a patient prior to performing OMT
- Use an -25 modifier when you bill an E&M (examination & medical) service the same day as the OMT
- Include a time designation in your record for the duration required to perform OMT
- Document the types of OMT you use to treat the patient (e.g. HVLA, muscle energy, MFR)
